# Pharmacological Treatment of Diabetic and Non-Diabetic Patients With Coronary Artery Disease in the Real World of General Practice

**DOI:** 10.3389/fphar.2022.858385

**Published:** 2022-03-24

**Authors:** Michelangelo Rottura, Antonino Molonia, Domenico Antonio Giorgi, Sebastiano Marino, Riccardo Scoglio, Giovanni Pallio, Natasha Irrera, Egidio Imbalzano, Domenica Altavilla, Giovanni Squadrito, Francesco Squadrito, Vincenzo Arcoraci

**Affiliations:** ^1^ Department of Clinical and Experimental Medicine, University of Messina, Messina, Italy; ^2^ Italian Society of General Practice (SIMG), Messina, Italy; ^3^ Department of Biomedical and Dental Sciences and Morphological and Functional Imaging University of Messina, Messina, Italy

**Keywords:** coronary artery disease, diabetes, pharmacological management, clinical practice, cardiovascular risk

## Abstract

Type 2 diabetes mellitus (T2DM) severely increases the probability of developing coronary artery disease (CAD), and diabetic patients with CAD should be considered at very high cardiovascular risk. The complexity of this clinical scenario makes very hard the appropriateness of the pharmacological treatment in the real world. To investigate the implementation of guideline recommendations for the treatment of patients affected by CAD with or without T2DM, a retrospective observational study was carried out between 2018 and 2020, by using the computerized clinical medical record of 10 general practitioners (GPs) including 13,206 subjects. A total of 926 patients (7.0%) were affected by CAD and 393 (42.4%) of them were also diabetic. LDLc, SBP, DBP, and FPG were recorded in 77.4%, 65.4%, 66.5%, and 82.6% of patients, respectively. Comorbidities (median; IQR = 8; 6–10 vs. 5; 3–7: *p* < 0.001) were significantly high in diabetic patients. Specialist counselling has been observed in 59.9% of diabetic and 57% of non-diabetic patients (*p* = 0.400). Antithrombotic drugs, statins, β-blockers, or RAASs were prescribed in 67.2%, 59.6%, and 75.9% of patients, respectively. Overall, 462 (49.9%) patients used the treatment suggested by guidelines. Dyslipidemia, hypertension, atherosclerosis, and specialist counselling were predictors of suggested drugs use both in diabetic and non-diabetic patients. Diabetes was not an independent factor related to the likelihood to be properly treated, according to the guidelines. Glucose lowering drugs were prescribed in 69.5% of diabetic patients, but only 39 (14.3%) were treated with the proper GLP-1 or SGLT2-i, whereas 45 patients (16.5%) received the improper sulphonylureas. Our results showed that a “non-ideal” therapeutic approach was adopted in patients affected by diabetes and CAD. ADA and ESC guidelines recommend the use of at least one hypoglycemic agent belonging to the GLP-1 or SGLT2-i class in diabetic patients with high/very high cardiovascular risk, regardless of the glycemic target (HbA1c <7%). However, only a few diabetic patients on hypoglycemic therapy were appropriately treated. These data suggest that a closer collaboration between the GPs, clinical pharmacologist, and specialists is needed in the real world scenario of the general practice in order to effectively improve adherence to guidelines and overall management of global cardiovascular risk in diabetic patients.

## Introduction

The prevalence of diabetes mellitus (DM) has rapidly increased in the last decade; DM is expected to affect more than 450 million individuals by 2025, thus causing a huge global burden for public health systems in terms of mortality, morbidity, related disability, and economical resource use (Saeedi et al., 2020).

Type 2 diabetes mellitus (T2DM) is a complex metabolic disease which multiplies the likelihood of developing macrovascular complication, such as coronary artery diseases (CAD) and peripheral arterial disease (PAD) (Shah et al., 2015; Joseph et al., 2020; Singh et al., 2021; Markousis-Mavrogenis et al., 2022), and microvascular complication, mainly nephropathy and retinopathy (Altavilla et al., 2009; Saputro et al., 2021; Xiong et al., 2021; Markousis-Mavrogenis et al., 2022).

Poor glycemic control is one of the main determinants of CVDs (Sardu et al., 2020), and in fact patients with T2DM are more susceptible to develop CAD, which in turn represents the main cause of death among patients suffering from this metabolic disease (Shah et al., 2015).

The pathophysiology of CAD is characterized by a complicated and not fully understood cascade of events involving several factors that are often linked and interconnected (Moreira et al., 2015; Medina-Leyte et al., 2021).

Over-inflammation, chronic or acute hyperglycemia, insulin resistance, hyperinsulinemia, and dyslipidemia in T2DM patients, together with decreased levels of high density lipoprotein (HDL) and increased levels of low density lipoprotein (LDL), represent the triggering causes of endothelial disfunction (i.e., impaired balance between the vasoconstriction and vasodilatory properties of endothelium) (Moreira et al., 2015; Medina-Leyte et al., 2021; Paolisso et al., 2021).

In addition, the increased production of reactive oxygen species (ROS) in T2DM patients causes vascular inflammation that also has an important role in CAD by amplifying the pathological loop that favors the spreading of atherosclerosis, endothelial dysfunction, and by prompting the development of plaque instability (Marfella et al., 2021).

Finally, a dangerous imbalance in the coagulation system may be also appreciated in this kind of patient, thus stimulating both the coagulation cascade and an exaggerated platelets aggregation. The clinical correlation of this complex pathophysiology is that patients generally require a close monitoring of the disease and an accurate and intensive pharmacological treatment, as specifically detailed in the recommendations issued by the European Society of Cardiology (Neumann et al., 2020). However, the implementation of the guidelines in the real world is not easy and it is frequently hindered by the presence of concomitant comorbidities, as previously reported (Rottura et al., 2021).

For all the above reasons, the aim of this study was to investigate the pharmacological management of diabetic and non-diabetic patients with CAD in the real world scenario of the general practice.

## Materials and Methods

### Data Sources

A retrospective cohort study was carried out using the computerized clinical medical record of a population of about 13,000 individuals living in the area of Messina (Sicily) and registered in the lists of 10 general practitioners (GPs).

GPs participating to this project agreed to record data during their daily clinical practice, through their dedicated clinical software, and to send complete and anonymous data about their patients to the unique central database. All GPs received extensive training in data collection procedure. Data quality checks were routinely performed through the analysis of several parameters, such as missing patient codes, number of daily filled prescriptions, and outliers. Any variation within defined ranges is investigated and back-submitted to each participating GP, in order to receive immediate feedback about data quality and completeness. Data quality and completeness have been already validated in previous drug-utilization studies (Barbieri et al., 2020; Squadrito et al., 2020; Rottura et al., 2021). The database contains the information recorded during 2018–2020 on each GP’s patient, aged at least 18 years old, including age, sex, height, weight and body mass index (BMI), systolic blood pressure (SBP) and diastolic blood pressure (DBP), information on lifestyle (alcohol, smoke), and data on diagnostic laboratory exams including fasting plasma glucose (FPG), glycated haemoglobin (HbA1c), and lipid profile. All drugs prescribed during the study period were recorded for each patient, as well as all morbidities verified since the registration date on the GP list. The anatomical therapeutic chemical (ATC) classification system was used to code information on drugs. Diagnoses were coded using the International Classification of Diseases, 9th Revision, Clinical Modification (ICD-9 CM).

### Study Population

All patients affected by CAD, as defined using ICD-9-CM coding (410*-414*), were selected. Among these, all diabetic patients (ICD9 code 250*) were identified. Moreover, the sub-population of patients affected by carotid artery disease (ICD9 code 433*) was identified.

Patients were followed until death, disenrollment, or end of the study, whichever occurred first.

A patient encrypted code has been used to maintain anonymity. The study protocol was approved by the local Ethical Committee of Messina University Hospital (n° prot. N.0010280/2020; Coordinator Centre).

### Data Analysis and Statistics

A descriptive analysis was performed to compare all the clinical and demographic characteristics of the study population among CDA patients, affected or not by diabetes.

Moreover, the sub-group of patients affected by carotid artery disease was also analysed.

Descriptive statistics were reported as medians, along with interquartile range (IQR), or absolute frequency and percentages, for continuous and categorical variables, respectively.

Because of a not normal distribution of some of the numerical variables, verified using the Kolmogorov–Smirnov test for normality, a non-parametric approach was adopted. The Mann–Whitney U test for independent sample and two-tailed Pearson chi-squared test were carried out to compare continuous and categorical variables, respectively.

Cohorts of CAD patients were described in terms of demographic (sex, age), comorbidities (identified at cohort entry), Charlson Comorbidity Index (CCI), therapeutic characteristics, and laboratory tests. Taking into account the very high cardiovascular risk of all CAD affected patients LDL-C, HbA1c and SBP-DBP target were also estimated, according to ESC guidelines (Cosentino et al., 2020). Lipid-lowering drug (LLD) prescriptions were identified using ATC code (C10AA*, C10BA*, C10AX09) and grouped as high intensity lipid-lowering drugs (rosuvastatin ≥20 mg, atorvastatin ≥40 mg; any statin plus ezetimibe) or low/moderate intensity lipid-lowering drugs.

High intensity lipid-lowering users were identified and stratified by targeting value. Adherence to therapy was calculated as medication possession ratio (MPR) with a cut-off of MPR ≥80. The Medication Possession Ratio (MPR) was calculated as the proportion of the number of tablets dispensed over the estimated period of LLD treatment.

The number of days covered by each patient was counted like the number of pills prescribed, assuming a single intake per day of LLD (Peterson et al., 2007). Since the number of prescriptions filled was used as a proxy for beneficiary status, users with a MPR >80% was established as threshold for adherence (Pittman et al., 2011).

Patients were stratified according to MPR and high intensity lipid-lowering drugs use and LDL target achievement analysed.

Glucose-lowering drugs (GLDs) prescriptions were identified using ATC code (A10A*, A10B*). Prevalence of GLDs use was measured for each drug class as the ratio between the number of patients who received at least one prescription of drug and the total number of diabetic patients. Treatments with sodium-glucose co-transporter 2 inhibitors (SGLT2-i) or Glucagon-like peptide-1 analogues (GLP-1), alone or in combination with other glucose-lowering drugs, were considered the recommended treatment, according to EAS/ESC guidelines (Cosentino et al., 2020).

Blood pressure-lowering drugs prescriptions were identified using ATC code (C02*, C03*, C07*, C08*, C09*). Number of different classes of drug as well as prevalence of use were evaluated.

According to the ESC guidelines, the use of β-blockers (BB) (C07*) and/or renin angiotensin aldosterone system inhibitors (RAASs) (C09*) plus statins plus antiplatelet drugs (B01AC*) were considered the recommended treatment to prevent events in CAD affected patients. Moreover, in atrial fibrillation (AF) affected patients Vitamin K antagonists (B01AA*) or new oral anticoagulants (NOAC) (B01AE*, B01AF*) other than antiplatelet drugs were taken into account.

Univariate logistic regression models were performed to identify predictors of recommended treatment use. All variables identified as predictors were included in a stepwise multivariate logistic regression model (backward procedure, α = 5%).

Univariate and multivariate logistic regression model were, also, performed to identify the factors associated with recommended glucose lowering drug use in diabetic patients, using patients without the suggested prescriptions as comparators.

Moreover, all variables not resulted significant in the univariate analysis, but considered clinically remarkable, and with a cut-off of alpha error ≤0.2 according to Hosmer–Lemeshow test, were also included (Sun et al., 1996; VanderWeele, 2019). Conversely, variables with the same clinically significant and with a plausible collinearity, verified by the Spearman’s rank correlation coefficient, were excluded from the multivariate model.

Odds ratios (ORs) with 95% confidence intervals (CIs) were calculated for each covariate of interest in univariate (crude OR) and multivariate (adjusted OR) regression models. The goodness of fit of the regression model was carried out by the Hosmer–Lemeshow test for adequacy.

A *p*-value < 0.05 was considered statistically significant. All statistical analyses were performed using SPSS version 23.0 (IBM Corp., SPSS Statistics, Armonk, NY, United States).

## Results

### Clinical Characteristics of the Study Population, Biochemistry Testing, and Comorbidities

A total of 926 patients (7.0%) out of 13,206 people covered by the medical care provided by 10 GPs were affected by CAD with a median age (IQR) of 74 years (65–83). Of them, 393 (42.4%) were also diabetic. No differences in age and gender were observed between groups. BMI, SBP, FPG, and HbA1c levels were significantly higher in diabetic patients, while LDL levels were significantly lower than non-diabetic patients (Table 1). During the 2 years of the study, at least one recorded value concerning LDLc, SBP, DBP, and FPG was observed in 77.4%, 65.4%, 66.5%, and 82.6% of CAD affected patients, respectively. Lifestyle habits, such as alcohol use or smoking status, and BMI were recorded in less than 50.0% of patients. However, clinical and laboratory tests were generally more frequently recorded in diabetic patients affected by CAD than in non-diabetic patients (Table 1). Hypertension (80.3%) and hyperlipemia (58.4%) were the most frequent comorbidities observed in CAD affected patients. Moreover, number of comorbidities as well as Charlson comorbidities index were significantly high in diabetic patients (Table 1). Specialist counselling was carried out in 539 (58.2%). No significant differences were observed between diabetic (59.9%) and non-diabetic (57.0%) patients (*p* = 0.400).

**TABLE 1:** Characteristics of diabetic and non-diabetic patients affected by CAD.

	DM patients	Non-DM patients	*p*-value	Total
	*N* = 393	*N* = 533		*N* = 926
Characteristics of patients				
Gender (M); N (%)	224 (57.1)	317 (59.5)	0.477	541 (58.5)
Age (years)	76 (66–83)	73 (63–83)	0.050	74 (65–83)
BMI; median (IQR)	29.4 (26.4–32.9)	27.2 (24.4–30.9)	<0.001	28.2 (25.2–32.0)
SBP; median (IQR)	135 (125–149)	130 (120–145)	0.011	135 (120–145)
DBP; median (IQR)	80 (70–80)	80 (70–80)	0.858	80 (70–80)
LDL-C; median (IQR)	88.5 (68–116)	99 (80–128)	<0.001	95 (74–122)
Total Cholesterol; median (IQR)	164 (135–199)	177 (150.3–204.8)	<0.001	172 (144–202)
HDL; median (IQR)	45 (37–53)	49 (43–59)	<0.001	47 (40–56)
Triglycerides; median (IQR)	120.5 (89–167.3)	104 (78–143.3)	<0.001	111.5 (82–156)
FPG; median (IQR)	123 (102–153.5)	95 (86–104)	<0.001	102 (90–122)
HbA1c; median (IQR)	6.8 (6–7.5)	5.7 (5.4–6)	<0.001	6.2 (5.7–7.2)
Different molecules; median (IQR)	17 (10–25)	12 (6–19)	<0.001	14 (8–21)
N. of prescriptions; median (IQR)	125 (57–184)	72 (25–121)	<0.001	93 (33–154)
Recording of laboratory values and lifestyle data				
BMI; N (%)	203 (51.7)	214 (40.2)	0.001	417 (45.0)
Smoking; N (%)	129 (32.8)	174 (32.6)	0.954	303 (32.7)
Alcohol use; N (%)	90 (22.9)	78 (14.6)	0.001	168 (18.1)
SBP; N (%)	269 (68.4)	337 (63.2)	0.099	606 (65.4)
DBP; N (%)	269 (68.4)	347 (65.1)	0.286	616 (66.5)
LDL-C; N (%)	326 (83.0)	391 (73.4)	0.001	717 (77.4)
Total Cholesterol; N (%)	339 (86.3)	428 (80.3)	0.017	767 (82.8)
HDL-C; N (%)	329 (83.7)	401 (75.2)	0.002	730 (78.8)
Triglycerides; N (%)	342 (87.0)	430 (80.7)	0.010	772 (83.4)
FPG; N (%)	333 (84.7)	432 (81.1)	0.144	765 (82.6)
HbA1c; N (%)	219 (55.7)	120 (22.5)	0.008	339 (36.6)
Comorbidity				
Arthritis and arthrosis; N (%)	217 (55.2)	237 (44.5)	0.001	454 (49.0)
Atherosclerosis; N (%)	95 (24.2)	90 (16.9)	0.006	185 (20.0)
Atrial fibrillation; N (%)	52 (13.2)	51 (9.6)	0.080	103 (11.1)
Cerebrovascular disease; N (%)	171 (43.5)	177 (33.2)	0.001	348 (37.6)
Chronic respiratory diseases; N (%)	205 (52.2)	231 (43.3)	0.008	436 (47.1)
CKD; N (%)	231 (58.8)	254 (47.7)	0.001	485 (52.4)
Dyslipidemia; N (%)	268 (68.2)	273 (51.2)	<0.001	541 (58.4)
Heart failure; N (%)	87 (22.1)	71 (13.3)	<0.001	158 (17.1)
Hypertension; N (%)	348 (88.5)	396 (74.3)	<0.001	744 (80.3)
Gout and metabolism disorders; N (%)	63 (16.0)	62 (11.6)	0.053	125 (13.5)
Neoplasm; N (%)	64 (16.3)	64 (12.0)	0.068	128 (13.8)
Obesity; N (%)	51 (13.0)	55 (10.3)	0.209	106 (11.4)
Osteoporosis; N (%)	135 (34.4)	142 (26.6)	0.011	277 (29.9)
Psychic sphere disorders; N (%)	197 (50.1)	242 (45.4)	0.155	439 (47.4)
CCI; N (%)	3 (2–5)	1 (0–3)	<0.001*	2 (1–4)
N. diseases; N (%)	8 (6–10)	5 (3–7)	<0.001	6 (4–8)

BMI, body mass index; CCI, charlson comorbidities index; CKD, chronic kidney disease; DBP, dyastolic blood pressure; DM, diabetes mellitus; FPG, fasting plasma glucose, HbA1c, glycated hemoglobin; HDL-C, high density lipoproteins cholesterol; IQR, inter quartile range; LDL-C, low-density lipoprotein cholesterol; M, male; N, number; SBP, systolic blood pressure.

### Pharmacological Management of CAD in Diabetic and Non-Diabetic Subjects

Antiplatelet drugs, or NOACS (in AF affected patients), were prescribed in 622 (67.2%) patients affected by CAD. In particular, diabetic patients (*n* = 283, 72.0%) showed a significant increase in antithrombotic agents use than that observed in non-diabetic patients (*n* = 339, 63.6%; *p* = 0.007).

Patients prescribed with statins were 552 (59.6%); treatment with statins was significantly adopted in diabetics (*n* = 251, 63.9%) compared to non-diabetic (*n* = 301, 56.5%) patients (*p* = 0.023). However, only 235 subjects (25.4%) used high intensity statins. In particular, 112 (44.6%) diabetic and 123 (40.9%) non-diabetic statin users were prescribed with high intensity statins (*p* = 0.374).

The achievement of LDL therapeutic target was observed in 58 (8.1%) patients. Diabetic (11.7%) patients significantly reached LDL target (*p* = 0.001) over non-diabetic (5.1%) patients. The probability to achieve LDL target was significantly higher in the group of patients adherent to high intensity statin treatment compared to not adherent low intensity statin users (OR 2.86; 95% CI 1.11–7.69: *p* = 0.029).

CAD affected patients prescribed with β-blockers (BBs) or Renin Angiotensin Aldosterone System inhibitors (RAASs) throughout the study period were 703 (75.9%): 312 (79.4%) diabetic patients and 391 (73.4%) not diabetic patients (*p* = 0.034).

Overall, almost half of CAD affected patients (462, 49.9%) were prescribed in agreement with the treatment suggested by the guidelines. The correct prescribing attitude was more frequent in diabetic (54.2%) than in non-diabetic (46.7%) patients (*p* = 0.024).

Hypertension was observed in 744 patients (80.3%). Of them, 517 (69.5%) had a recorded blood pressure value and only 281 (54.4%) patients out of 517 reached the recommended blood pressure target.

Among hypertensive patients, 634 (85.2%) were treated with blood pressure lowering drugs and 13.6% of them were on monotherapy. No statistical difference was observed between diabetic and non-diabetic patients in drug use (84.2% vs. 86.1%, *p* = 0.463), blood pressure recording (71.3% vs. 67.9%, *p* = 0.324), or blood pressure target achievement (51.6% vs. 56.9%, *p* = 0.230). In addition, a significant increase in monotherapy use was observed in non-diabetic patients over in diabetic ones (16.1% vs. 10.6%, *p* = 0.042).

Male patients affected by dyslipidemia, hypertension, or atherosclerosis were more likely to be prescribed with guidelines recommended treatment both in diabetic and non-diabetic patients. Moreover, the probability to be treated with recommended drugs increases only in diabetic patients when they were also affected by cerebrovascular disease (OR = 3.60, 95% CI 2.22–5.82).

In addition, the likelihood to be treated according with the guidelines increased with the number of other drugs used and with, at least, one specialist counselling during the study. Conversely, CKD, chronic respiratory diseases, and mood disorders were correlated with a low probability of being correctly treated. In CAD affected patients, diabetes was not an independent factor related to the likelihood to be properly treated, according to the guidelines (Table 2).

**TABLE 2:** Predictive factors of recommended treatment use in CAD affected patients.

	Crude OR [IC95%]	*p*-value	Adjusted OR [IC95%]	*p*-value
Gender, (M)	2.17 (1.66–2.83)	<0.001	2.07 (1.49–2.88)	<0.001
Age (years)	1.01 (1.00–1.02)	0.082	1.01 (1.00–1.02)	0.159
Lifestyle				
BMI	0.99 (0.96–1.02)	0.495		
Alcohol abuse	1.03 (0.50–2.09)	0.945		
Smoking	0.85 (0.51–1.44)	0.549		
Comorbidity				
Neoplasm	1.30 (0.89–1.89)	0.175	1.14 (0.72–1.80)	0.584
Dyslipidemia	2.48 (1.89–3.24)	<0.001	2.67 (1.91–3.73)	<0.001
Gout and metabolism disorders	1.28 (0.88–1.87)	0.203		
Heart failure	1.04 (0.74–1.46)	0.838		
Hypertension	1.78 (1.28–2.48)	0.001	1.64 (1.10–2.48)	0.018
Atrial fibrillation	0.72 (0.48–1.09)	0.124	0.65 (0.39–1.08)	0.094
Cerebrovascular disease	1.26 (0.96–1.64)	0.093	1.07 (0.76–1.52)	0.703
Atherosclerosis	1.87 (1.34–2.60)	<0.001	1.76 (1.18–2.62)	0.005
CKD	0.72 (0.56–0.93)	0.013	0.66 (0.47–0.94)	0.021
Chronic respiratory diseases	0.61 (0.47–0.79)	<0.001	0.39 (0.28–0.55)	<0.001
Obesity	1.14 (0.76–1.71)	0.520		
Diabetes Mellitus	1.35 (1.04–1.75)	0.025	0.85 (0.61–1.18)	0.340
Psychic sphere disorders	0.57 (0.44–0.74)	<0.001	0.51 (0.37–0.72)	<0.001
Osteoporosis	0.72 (0.54–0.95)	0.020	0.72 (0.48–1.08)	0.110
Arthritis and arthrosis	0.88 (0.68–1.14)	0.324		
Number of diseases	1.02 (0.98–1.07)	0.308		
Different molecules	1.08 (1.06–1.09)	<0.001	1.03 (1.00–1.06)	0.064
Number of prescriptions	1.01 (1.01–1.02)	<0.001	1.01 (1.00–1.01)	<0.001
CCI	1.03 (0.97–1.09)	0.314		
Specialist counselling	4.10 (3.10–5.43)	<0.001	3.07 (2.22–4.25)	<0.001

BMI, body mass index; CCI, charlson comorbidity index; CI, confidence interval; CKD, chronic kidney disease; M, male; OR, odds ratio; IQR, interquartile range; M, male.

CAD patients with concomitant carotid artery disease were 168 (18.1%). Carotid artery diseases were mainly observed in diabetics over non-diabetic patients (24.6% vs. 15.6%, respectively) (*p* = 0.018).

The 64.9% of patients with carotid artery diseases were prescribed in agreement with the treatment suggested by the guidelines. In addition, the probability to be properly treated significantly increased in this group of patients (adj. OR = 1.78; 95% CI 1.16–2.74), as well as the antiplatelet drug use, alone [adj. OR (95% CI) = 1.98 (1.16–3.37)].

Furthermore, among patients with carotid artery diseases, 76.5% of diabetic and 53.0% of non-diabetic patients (*p* = 0.001) were treated in accordance with guidelines recommendation.

Patients suffering from carotid artery obstruction and affected by diabetes were more likely to be properly treated compared to non-diabetic patients [adj. OR (95% CI) = 5.07 (2.18–11.81)]. Equally, the probability to be treated with antiplatelet drugs (adj. OR = 3.44; 95% CI 1.15–10.25) and lipid lowering drugs (adj. OR = 3.99; 95% CI 1.50–10.62) significantly increased in this group of patients.

### Pharmacological Management of Diabetes in Patients With CAD

Among the 393 patients affected by CAD and diabetes, only 55.7% had at least one HBA1c value recorded in the 2 year study period. Of them, 45.2% were out of therapeutic target (>7%; >53 mmol/L). Glucose lowering drugs were prescribed in 273 patients (69.5%), but only 39 diabetics (14.3%) were treated with the proper GLP-1 or SGLT2-i, whereas 45 patients (16.5%) received the improper sulfonylureas. Metformin (72.2%) was the most used hypoglycemic drug (Table 3). In addition, among all factors analyzed, only diabetic patient age was inversely correlated with GLP-1 or SGLT2-i use (OR: 0.94, 95% CI 0.91–098).

**TABLE 3:** Classes of glucose lowering drugs (ATC level IV) used by patients with CAD and diabetes.

ATC level	Total & *N* = 273 (%)
A10A-Insulins and Analogues	108 (39.6)
A10BA-Biguanided	166 (60.8)
A10BB-Sulfonylureas	40 (14.7)
A10BD- Combinations of oral blood glucose lowering drugs	31 (11.4)
A10BF- Alpha glucosidase inhibitors	25 (9.2)
A10BG-Thiazolidinediones	2 (0.7)
A10BH-Dipeptidyl peptidase 4 (DPP-4) inhibitors	20 (7.3)
A10BJ-Glucagon-like peptide-1 (GLP-1) analogues	20 (7.3)
A10BK-Sodium-glucose co-transporter 2 (SGLT2) inhibitors	13 (4.8)
A10BX- Other blood glucose lowering drugs, excl. insulins	62 (22.7)

ATC, Anatomical Therapeutic Chemical Classification System. &Drug classes use was not mutually exclusive.

## Discussion

A large proportion of people worldwide is affected by diabetes and the number of patients suffering from this disease is expected to rapidly increase in the next few years. The diagnosis of T2DM severely augments the probability of developing CAD and, as a consequence, the cardiovascular disease is the main cause of morbidity and mortality in the diabetic population. To mitigate this very high cardiovascular disease burden it is of paramount importance to curb effectively the multiple risk factors that cooperate and synergize together in the speeding and worsening of cardiovascular complications. These risk factors include genetic background, dyslipidemia, hypertension, and hyperglycemia that, together with diabetes, are often frequent in patients of advanced age (Kannel, 2002; Nesto, 2005; The Diabetes Control and Complications Trial/Epidemiology of Diabetes Interventions and Complications (DCCT/EDIC) Study Research Group, 2005; Goff et al., 2007; Mourad and le Jeune, 2008; The ADVANCE Collaborative Group, 2008; Ahlqvist et al., 2015). Moreover, age is the strongest factor related to the development of CAD which is a leading cause of morbidity and mortality in older adults (Benjamin et al., 2017).

The complexity of this clinical scenario makes generally very hard the appropriateness of the pharmacological treatment in the real world, as previously shown (Squadrito et al., 2020; Rottura et al., 2021). An optimal management of diabetes, hypertension, and other cardiovascular risk factors in patients with CAD is essential, especially in the elderly. Indeed, the best pharmacological management is recommended by the scientific societies that periodically issue, in light of the evident available clinical proofs, appropriate guidelines to address the clinical and therapeutic needs. The aims are to make patient management by the physicians easier and to render available to all patients the most updated and accurate treatment.

Theoretically the transfer process in the general practice might be simple, but it very often encounters difficulties and barriers in the general practice. Furthermore, the implementation of the guidelines comes from physician personal judgement on the clinical status of the patient but, sometime, the whole therapeutic management also needs clinical specialist involvement. Finally, the presence of comorbidities and polytherapy may further complicate patient management.

More specifically, T2DM increases about two-fold the risk for coronary heart disease and worsens the outcome of the patients with CAD; moreover, in elderly diabetic patients a close risk was observed for ischemic as well as for hemorrhagic stroke, and for other vascular deaths (The Emerging Risk Factors Collaboration, 2010). For this reason, a close attention to T2DM that multiplies the cardiovascular risk is required.

The most recent recommendation on the pharmacological treatment of CAD states that patients should be treated with aspirin and eventually a non-vitamin-K antagonist oral anticoagulant (NOAC), if atrial fibrillation is present, lipid lowering drugs, BBs, and RAAS inhibitors (Neumann et al., 2020). In addition, because of very high cardiovascular risk, CAD patients with diabetes mellitus should receive a sodium-glucose co-transporter 2 inhibitors or a glucagon-like peptide-1 receptor agonist (Cosentino et al., 2020).

Indeed, SGLT2 inhibitors and GLP1 agonists play a key role in the modulation of diabetes-related dysfunction. Thus, the block of SGLT2 pathways could interfere with different and multiple pathways via best glycemic control and significant reduction of inflammation and endothelial dysfunction in high risk patients, as those affected by T2DM and multi-vessel coronary stenosis. Indeed, the SGLT2 receptors are over-expressed in humans’ districts with higher inflammatory/oxidative stress burden and contributes to endothelial dysfunction and cardiovascular disease (D’Onofrio et al., 2021). Moreover, GLP-1 axis may play a protective role in patients with T2DM. Indeed, as recently seen, the therapeutic use of GLP-1 analogous decreases the number of hospitalizations and improves the prognosis of diabetic patients through a reduction of over-inflammation and metabolic distress (Sardu et al., 2018).

To investigate the adhesion to guideline recommendations of CAD patients with or without T2DM in the real scenario of general practice, a retrospective cohort study was performed by analysing the computerized clinical medical record of 10 GPs, thus including 13,206 patients. The results suggest that GPs did not adequately assess the clinical status of their patients with CAD. Those subjects are included in the high/very high cardiovascular risk class, most of them are elderly, and they should be monitored with close attention. Indeed, such patients should maintain or reach the target values defined by the European cardiology guidelines (LDL <55 mg/dl; SBP> 120 and <140 mmHg—DBP <80 mmHg, HbA1c <7%). The concomitant presence of diabetes improved monitoring of CAD patients. In fact, the recording frequency of all laboratory data (with the exception of blood pressure) and lifestyle data (except smoking) were higher in patients with diabetes than in non-diabetic patients. However, laboratory data monitoring could be further improved.

Furthermore, despite large scientific evidence that has pointed out the benefits of the treatment recommended by the European guidelines in reducing the risk of developing cardiovascular events, about half of patients with CAD were not yet adequately treated. Diabetic patients were better managed by GPs, at least from the point of view of the pharmacological treatment. Indeed, a significantly higher frequency of patients prescribed with the recommended therapy was observed in diabetics than in nondiabetics. Furthermore, even taking in consideration the single treatments (lipid-lowering, anti-ischemic, antithrombotic), diabetic patients were more adequately treated than non-diabetics.

Interestingly, this greater emphasis and attention on the prescriptions of the recommended therapy by GPs do not seem to be due to the diagnosis of diabetes, but it is more likely linked to the concomitant presence of other comorbidities that are generally more frequent in diabetic patients than in non-diabetic patients (i.e., hypertension, dyslipidemia, atherosclerosis, etc.). In fact, the logistic regressions analysis clearly show that diabetes was not an independent predictor of being treated in agreement with the recommended guidelines.

However, when the specific sub-group of patients suffering from carotid artery obstruction was considered, diabetes clearly increased the probability to be properly treated with antiplatelet drugs and lipid lowering drugs. Probably, in elderly patients identified with even high risk of cerebrovascular events, GPs pay more attention to the two-fold additional risk induced by diabetes and treat more accurately.

Generally, GPs seem to treat the single comorbidity more than the overall cardiovascular risk and only specialist counseling was found to be a determining factor in improving the therapeutic management of patients with CAD. In fact, logistic regression analyses confirmed that specialist counselling was an independent predictor of receiving an adequate treatment, as suggested by the ESC guidelines. However, more than 40% of patients never underwent specialist counselling. Too-long waiting lists for specialist consultations as well as the lack of a direct collaboration between GPs and specialists could justify this observation (De Luca et al., 2005).

The poor attention paid by the GPs to prescribe treatments aimed at reducing organ damage is further suggested by the “non-ideal” hypoglycemic therapeutic approach observed in patients with diabetes and CAD. In fact, in the light of recent scientific evidence, both ADA and ESC guidelines recommend the use of at least one hypoglycemic agent belonging to the GLP-1 or SGLT2-i class in diabetic patients with high/very high CV risk, regardless of the glycemic target (HbA1c <7%) to be achieved (Cosentino et al., 2020; Mach et al., 2020). Surprisingly, only the 14.3% of diabetic patients using GLDs were treated appropriately.

However, the increasing evidence on CV morbidity and mortality reduction in very high risk T2DM patients treated with SGLT2-i or GLP-1 was only recently considered and adopted in the last guidelines version. As a consequence, these are not yet so extensively applied by all physicians. Moreover, in Italy, GPs can prescribe SGLT2-i or GLP-1 only after a specific approval from the specialist. Surely, a more collaborative approach between GPs and clinical specialists is needed to improve the management of CAD affected diabetic patients.

Finally, the optimal use of drugs surely provides significant benefit for elderly patients with CAD, such as the population we analyzed. However, the age-related changes in body composition and hepatic and renal function could modify the bioavailability, metabolism, and elimination of drugs. In addition, the need to use several medications increases the risk of drug-drug interactions and adverse drug reactions, as well as non-adherence or drug discontinuation (Fleg et al., 2011; Rossello et al., 2015).

As a consequence, these data collectively suggest that in the real world scenario of the general practice there is a strong need of a closer collaboration between the GPs and clinical pharmacologist to effectively improve adherence to guidelines and overall management of global cardiovascular risk in diabetic patients.

## Data Availability

The raw data supporting the conclusion of this article will be made available by the authors, without undue reservation.
